# Supplementary material for: Low Back Pain Prevalence among Distance Learning Students
Source: Int J Environ Res Public Health. 2022 Dec 26;20(1):342. doi: 10.3390/ijerph20010342 (PMC9819147; doi:10.3390/ijerph20010342)
Supplement: Supplementary file 1 [file ijerph-20-00342-s001.zip › ijerph-2089986-supplementary.pdf]

Supplementary file: Research questionnaire with open end questions

Q1- sex
Q2- the age
Q3- height
Q4- weight
Q5- academic level
Q6- Did you exercise before the online education period?
Q7- Did you exercise while studying online?
Q8- How many hours did you spend on electronic devices during the online study period?
Q9- Do you suffer from chronic diseases?
Q10- Did you suffer from pain in the lower back before the online study period?
Q11- Have you had a lower back injury?
Q12- How much was the pain in the lower back area (note 1 almost no pain - 10 worst pain you've seen in your life) before an online study?
Q13- How much was the pain in the lower back area (note 1 almost no pain - 10 worst pain you've seen in your life) during an online study?
Q14- How much was the pain in the lower back area (note 1 almost no pain - 10 worst pain you've seen in your life) after an online study?
Q15- What is the nature of pain?
Q16- The nature of the pain you feel
Q17- Was the pain restricting movement?
Q18- Do you take any pain relief medication?
Q19- Have you had corona virus?
Q20- Do you think the corona virus has anything to do with this?
Q21- Did the pain continue after being infected with the corona virus?
Q22- Is lower back pain affected by corona virus?
Q23- Did you suffer from psychological stress while studying at a distance?
Q24- How many hours did you sleep today, approximately during the distance study period?
Q25- What was the position in which you were watching (lessons / lectures)
Q26- Have you gone to physical therapy because of lower back pain?
